# Semi-automated Anatomical Labeling and Inter-subject Warping of High-Density Intracranial Recording Electrodes in Electrocorticography

**DOI:** 10.3389/fninf.2017.00062

**Published:** 2017-10-31

**Authors:** Liberty S. Hamilton, David L. Chang, Morgan B. Lee, Edward F. Chang

**Affiliations:** ^1^Department of Neurosurgery, University of California, San Francisco, San Francisco, CA, United States; ^2^Center for Integrative Neuroscience, University of California, San Francisco, San Francisco, CA, United States

**Keywords:** intracranial recordings, electrode localization, electrocorticography, epilepsy, surgery, image coregistration, subdural electrodes, open science

## Abstract

In this article, we introduce img_pipe, our open source python package for preprocessing of imaging data for use in intracranial electrocorticography (ECoG) and intracranial stereo-EEG analyses. The process of electrode localization, labeling, and warping for use in ECoG currently varies widely across laboratories, and it is usually performed with custom, lab-specific code. This python package aims to provide a standardized interface for these procedures, as well as code to plot and display results on 3D cortical surface meshes. It gives the user an easy interface to create anatomically labeled electrodes that can also be warped to an atlas brain, starting with only a preoperative T1 MRI scan and a postoperative CT scan. We describe the full capabilities of our imaging pipeline and present a step-by-step protocol for users.

## Introduction

High-density electrocorticography (ECoG) is an invasive method where recordings are obtained directly from the surface of the brain in patients with medically intractable epilepsy. This approach provides millimeter spatial and millisecond temporal resolution neurophysiological data from awake, behaving humans, which complements the information obtained from noninvasive approaches such as, fMRI, EEG, and MEG (Chang, 2015). Preprocessing of ECoG data typically relies on aligning a preoperative MRI scan to a postoperative CT scan or postoperative MRI, then electrodes are localized either manually or in a semi-automated fashion (Kovalev et al., 2005; Miller et al., 2007; Dalal et al., 2008; Hermes et al., 2010; Oostenveld et al., 2011; Dykstra et al., 2012; Yang et al., 2012; Groppe et al., 2016; LaPlante et al., 2016). Once electrodes are localized in the MRI, they are assigned anatomical labels, and then potentially warped to a common MNI atlas space for comparisons across subjects. While many labs that perform ECoG research have their own methods for performing these steps, to our knowledge there exists no software package that incorporates all steps of image processing necessary for ECoG electrode localization and warping from start to finish.

Here, we present a protocol to perform all of these steps, from pial surface reconstruction to CT coregistration, electrode identification and anatomical labeling, and warping to a common atlas space. We take advantage of tools provided in the nipy software package (https://github.com/nipy/nipy/), dural surface reconstruction from ielu (LaPlante et al., 2016), 3D plotting in mayavi (http://mayavi.sourceforge.net/; Ramachandran, 2001), and extend on functions available in the MATLAB-based CTMR package (Hermes et al., 2010). This protocol has been used to localize and label electrodes in our previously published work (Dichter et al., 2016; Hamilton et al., 2016; Leonard et al., 2016; Moses et al., 2016; Muller et al., 2016b; Tang et al., 2017). In an effort to promote open and affordable access to these tools, all requirements to run the pipeline (aside from physical hardware) are freely available for download at no cost to the user. We hope that this software will facilitate more efficient workflows within ECoG research labs and will aid in reproducibility across studies.

## Materials and methods

### Subjects

Here, we present electrode localizations from human subjects undergoing surgical treatment for intractable epilepsy. Subjects were implanted with high-density subdural intracranial electrode grids (AdTech 256 channels, 4 mm center-to-center spacing and 1.17 mm diameter), subdural electrode strips (1 cm spacing), and/or depth electrodes (5 mm spacing) as part of their clinical evaluation for epilepsy surgery. This study was carried out in accordance with the recommendations of the University of California, San Francisco Institutional Review Board with written informed consent from all subjects. All subjects gave written informed consent in accordance with the Declaration of Helsinki. This protocol was approved by the University of California, San Francisco Institutional Review Board.

### Example data

**Figure 1:**
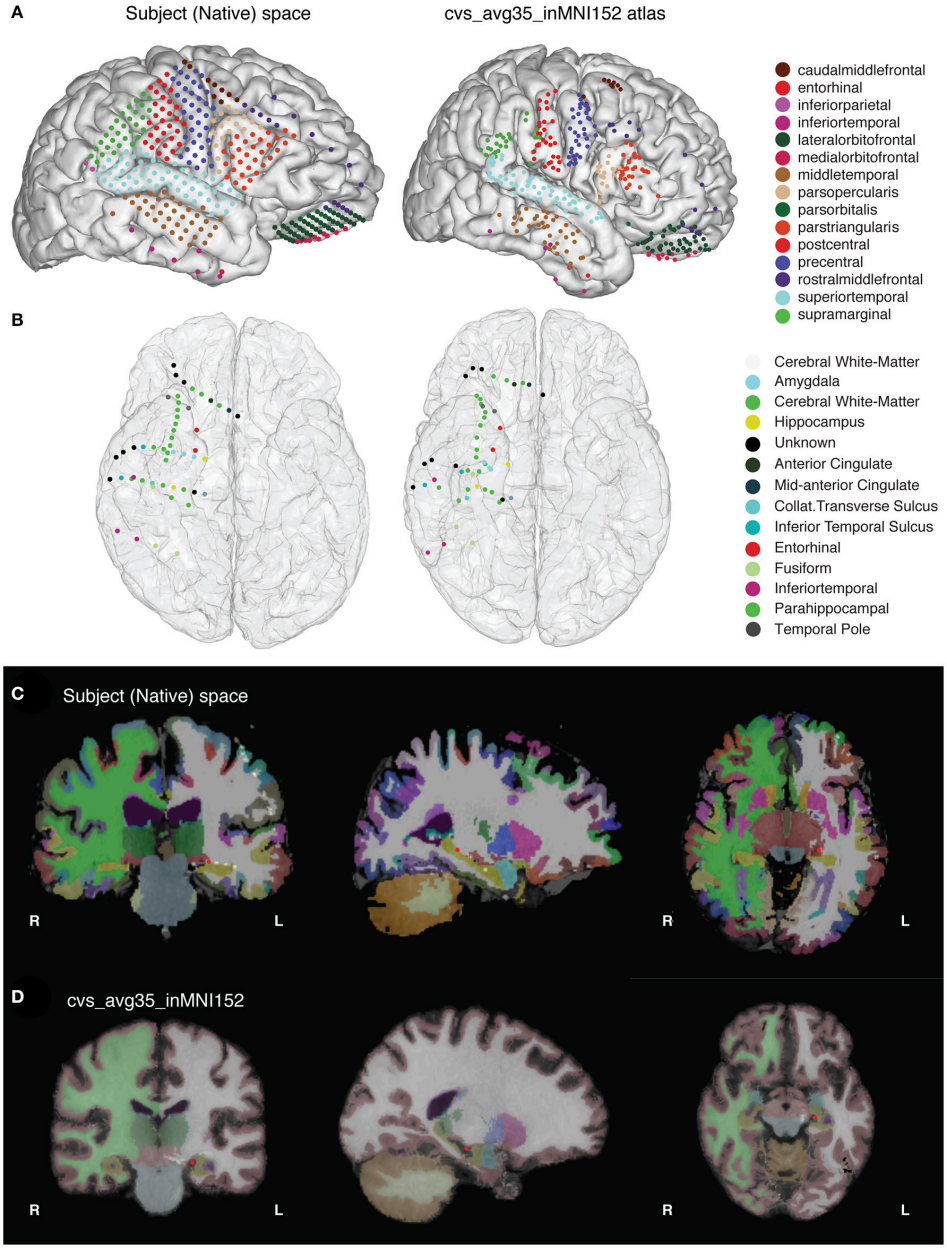
**(A)** The final results of the imaging pipeline are **(A)** anatomically labeled surface electrodes in the subject's native space (left) and in nonlinearly warped atlas space (right) and **(B)** anatomically labeled depth electrodes in subject (left) and atlas (right) space. **(C,D)** show segmented, labeled MRI volumes from freesurfer in the native **(C)** and atlas **(D)** space, with one example electrode in the hippocampus identified in red.

We include sample data for use with this pipeline so that the user may follow along with each of these steps and check their results. Sample data is available at https://doi.org/10.5281/zenodo.996813 and includes an AC-PC aligned T1 MRI scan, CT scan, and all intermediate and final files from the execution of img_pipe. This subject's data is shown in Figure 1A. Other figures include data from this subject and others to illustrate a wide variety of scenarios that could be encountered when using our software. We suggest that users who wish to follow along download this data set, then copy the files from the acpc and CT directories to a new Freesurfer subject directory. The user can then start from the prep_recon() step. The montage and electrode device details are specified in the dataset README file and accompanying montage file.

### Operating system requirements

1. Computer running Linux or Mac OS X. (Windows users should run this code on a virtual machine running Linux—plotting code will work natively on Windows, but freesurfer will not.)
2. Processor speed: at least 2 GHz
3. RAM: 8 GB or higher recommended
4. Graphics card (optional): 3D graphics card and accelerated OpenGL drivers

### Installation and third-party software requirements

In order to use the software described in this paper, the user will need to install the following third-party software packages:

1. gcc compiler (for Apple, can be downloaded through Apple Developer Command Line tools in XCode) or C++ compiler (available for Windows at http://aka.ms/vcpython27)
2. Anaconda Python 2.7 or 3.5 (we recommend Anaconda python for ease of installation https://www.continuum.io/downloads). Python 3.6 is not currently supported.
3. conda installer (included with Anaconda python installation)
4. Freesurfer (https://surfer.nmr.mgh.harvard.edu/fswiki/DownloadAndInstall). Be sure to register and copy the license.txt file to the appropriate directory. If the user is running Windows, they will have to run Freesurfer through a virtual machine running Linux (see https://surfer.nmr.mgh.harvard.edu/fswiki/Installation/FreeSurferVirtualImage).
5. For warping depth electrodes, libboost C++ libraries (v1.41) (http://www.boost.org/users/history/version_1_41_0.html).
6. Optional: If using an NVIDIA graphics card, some computations can be sped up by downloading and installing the CUDA libraries (we have found that CUDA v5.5 works with Freesurfer)
7. For converting dicom to niftii, either use SPM12 or use dcm2nii binary (https://www.nitrc.org/plugins/mwiki/index.php/dcm2nii:MainPage).

After installing the software above, the img_pipe module is installed by running the following commands at the terminal. We also suggest installing packages in a conda environment to avoid conflicts with any other installed software.

For Python 2.7, using conda located in your Python 2.7 installation directory:


$ git clone https://github.com/changlabucsf/img_pipe
$ conda env create -f img_pipe/environment
  _py27.yml
$ source activate img_pipe_py2
$ ipython
$ import img_pipe


For Python 3.5, using conda located in your Python 3.5 installation directory:


$ git clone https://github.com/changlabucsf/img_pipe
$ conda env create -f img_pipe/environment
  _py35.yml
$ source activate img_pipe_py3
$ ipython
$ from img_pipe import img_pipe


## Results and stepwise procedures

### Overview of the image preprocessing and electrode localization pipeline

This paper describes how to use the img_pipe package, which will allow the user to take a T1 structural MRI scan and a CT scan with intracranial electrodes from the same patient, identify electrodes for visualization on the pial surface, automatically label electrodes with anatomical labels according to the Desikan-Killany atlas (Desikan et al., 2006) and/or Destrieux atlas (Fischl et al., 2004), and nonlinearly warp electrodes onto a common atlas brain while preserving their anatomical locations. Results of this procedure are shown in Figure 1.

**Figure 2:**
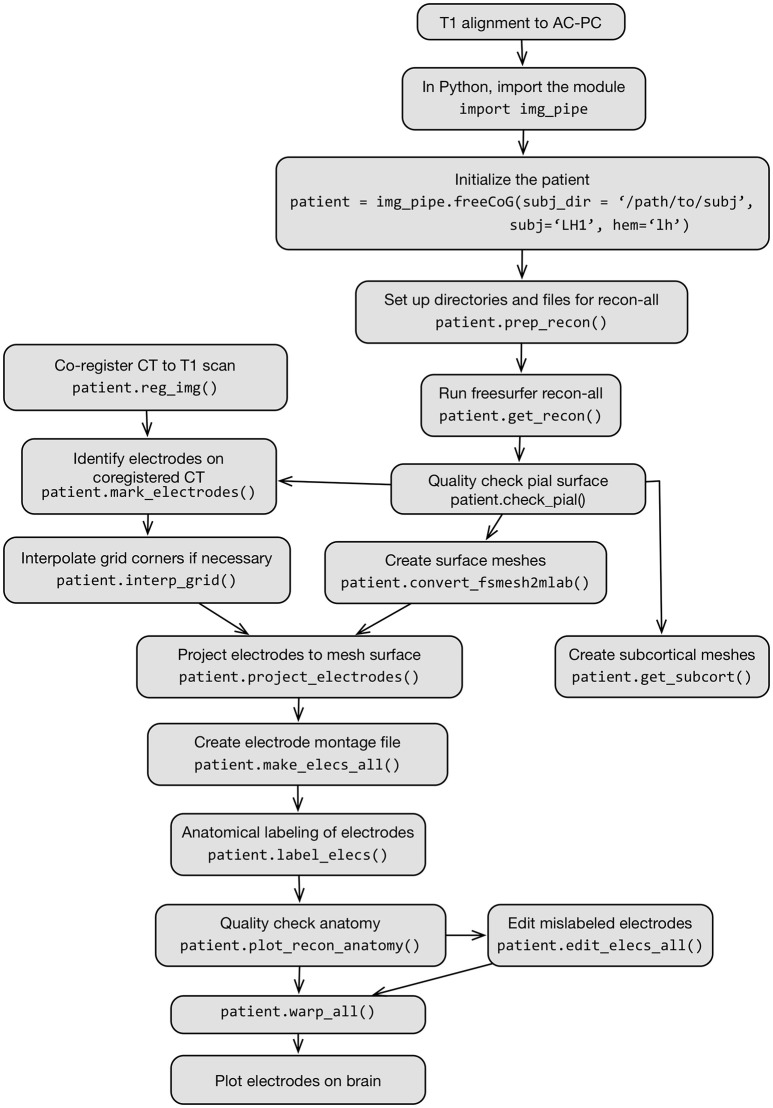
Flow chart schematic of imaging pipeline and use of the class img_pipe.freeCoG for electrode localization, anatomical identification, and warping.

The full process is described in Figure 2, which shows a flow chart of each step in this protocol.

### Setting up the directory structure and paths

- Set up the paths. If using the bash shell, edit ~/.bashrc, ~/.bash_profile to add the following lines:
   
  $ export FREESURFER_HOME="/path/to/
      freesurfer"
  $ source $FREESURFER_HOME/
      SetUpFreeSurfer.sh
  $ export SUBJECTS_DIR="/path/to/
      freesurfer/subjects"
  $ export
      DYLD_FALLBACK_LIBRARY_PATH="/usr/
      lib:$DYLD_LIBRARY_PATH"
  For example FREESURFER_HOME might be /Applications/freesurfer/ or /usr/local/freesurfer; SUBJECTS_DIR is a directory of the user's choosing but is often /usr/local/freesurfer/subjects or /Applications/freesurfer/subjects.
- After adding these lines to ~/.bashrc or ~/.bash_profile and saving the file, be sure to run:
  $ source ~/.bash_profile
  in the terminal. This will run these commands to set the appropriate environmental variables for use by img_pipe and Freesurfer.
- Running img_pipe requires a good quality, high resolution (preferably 1 mm isotropic) pre-operative T1 structural scan and a post-operative CT scan for the patient, both in nifti format. For specific requirements regarding T1 pulse sequences that work best, consult the Freesurfer beginners guide (https://surfer.nmr.mgh.harvard.edu/fswiki/FreeSurferBeginnersGuide). In general, a Siemens MPRAGE or GE SPGR sequence with excellent gray/white matter contrast will work well.
- After downloading and installing the packages described above, create a new subject directory with the patient ID (for this paper, we will use the example ‘test_subj’) in the freesurfer subjects directory (for example /usr/local/freesurfer/subjects/test_subj). In a terminal, run:
  $ mkdir $SUBJECTS_DIR/test_subj
- In this directory, create the directories acpc and CT:
  $ mkdir $SUBJECTS_DIR/test_subj/acpc
  $ mkdir $SUBJECTS_DIR/test_subj/CT
- Place the niftii format T1 scan in the acpc directory, and the niftii format CT scan in the CT directory. The T1 scan should be named T1_orig.nii and the CT scan should be named CT.nii.

### Alignment of T1 scan to AC-PC axis

It is recommended that the user align T1 scans to the anterior commissure-posterior commissure axis before processing in Freesurfer. This will provide better initial conditions for later warping steps, and will result in the creation of meshes in a standard orientation. While some programs are able to perform this alignment automatically (e.g., the Automatic Registration Toolbox, available as a linux command line tool: https://www.nitrc.org/projects/art), here we describe how to perform this process manually. Note: it is also possible to perform this alignment on the final surfaces and electrode files using Freesurfer's talairach.xfm file—see Section Other Notes for how to do this.

**Figure 3:**
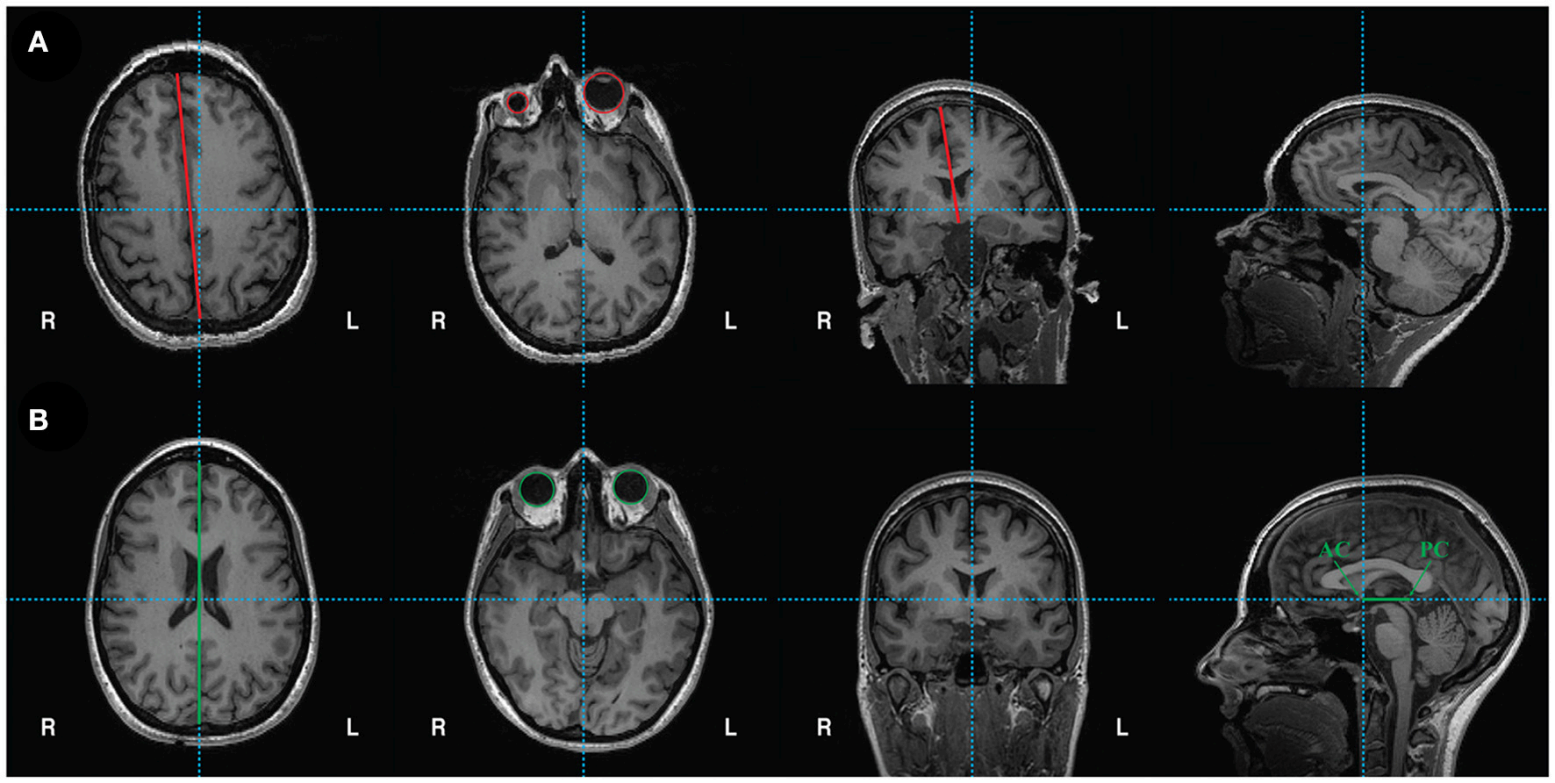
**(A)** Unaligned T1 scan. From left to right: axial view shows vertical crosshair unaligned with longitudinal fissure, which can be corrected by adjusting yaw. Next, another axial view depicts unequal size in eyes, which can be corrected by adjusting roll. Coronal view shows vertical crosshair unaligned with longitudinal fissure, which can also be corrected by adjusting roll. Lastly, the sagittal view shows that the horizontal crosshair is not aligned with the anterior commissure (AC) and posterior commissure (PC), which can be fixed by adjusting pitch. **(B)** Reoriented, ACPC aligned T1 scan with corrections in yaw, roll, and pitch. From left to right: axial view shows crosshairs aligned with longitudinal fissure. Another axial view shows equal sized eyes. Next, coronal view shows vertical crosshair aligned with longitudinal fissure. Finally, sagittal view shows the anterior commissure (AC) and posterior commissure (PC) aligned on the horizontal axis. The origin (0, 0, 0) is set to the anterior commissure.

- Alignment of the T1 scan to the anterior commissure-posterior commissure axis is performed in Freeview (Figure 3). Open Freeview and load the unaligned T1 scan in the Volumes tab. To aid in axis alignment, change the cursor style “long” in Preferences 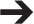 Cursor style “Long.” The color may also be changed if desired.
- To adjust the rotation and translation of the image, select Tools 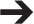 Transform Volume. Adjust the roll (with Y (P-A)) and yaw (with Z (I-S)) as necessary to make sure the head is aligned. Check the axial view to make sure the eyes show equally in the same slice (see Figure 3A vs. Figure 3B, second panel for unaligned and aligned examples). Make sure the midsagittal line is vertical in the axial view (see Figure 3A vs. Figure 3B, first and third panels) and in the coronal view. Choose Sample method “Cubic”.
- Select the anterior commissure and adjust the pitch of the head so that it is in line with the posterior commissure on the horizontal axis (Figures 3A,B, last panel).
- Finally, move to the (0, 0, 0) RAS coordinate (not TkReg RAS, just RAS). In the Transform Volume tool, translate the image until the cursor is at the anterior commissure.
- Once the brain is in a good orientation, click “Save Reg…” and save the transformation matrix in the acpc directory as T1_reorient.lta. Then, click “Save as…” and save the reoriented T1 file as T1.nii in the acpc directory (e.g. /usr/local/freesurfer/subjects/test_subj/acpc).

### Overview of the class freeCoG

- The img_pipe.py python module centers around the use of the class freeCoG, which is first initialized to contain information about a specific patient's data and has a number of methods that can be called to perform image coregistration, pial surface extraction, electrode anatomical labeling, and warping of electrodes into common space.
- The freeCoG class contains the following attributes that should be specified during initialization:
  ◦ subj: [string] the name of the subject ID. In this protocol, we will use 'test_subj' as the subject ID.
  ◦ hem: [string], 'lh', 'rh', or 'stereo'. The hemisphere of implantation (can also be stereo for bilateral coverage)
  ◦ zero_indexed_electrodes: [boolean, default = True], if False, will use one-indexed numbering for electrodes. This is important to note if your montage starts with electrode channel 1 vs. electrode channel 0 (since zero-indexing is the python default).
- The following should be specified during initialization if not already in the user's path. By default they will be set to the value in the environmental variables defined previously.
  ◦ subj_dir: [string]: specify the location of the freesurfer $SUBJECTS_DIR
  ◦ fs_dir [string]: the directory containing the Freesurfer executables, e.g., ‘/Applications/freesurfer’
- The class freeCoG also contains the following methods:
  ◦ prep_recon()
    ■ This method sets up the directory structure before running the Freesurfer pipeline.
  ◦ get_recon()
    ■ This method starts the Freesurfer recon-all pipeline, which takes a T1 scan and performs automatic extraction of the pial surface.
  ◦ convert_fsmesh2mlab()
    ■ This method converts the Freesurfer pial surfaces to triangle-mesh format for use/visualization in MATLAB and python.
  ◦ reg_img()
    ■ This method registers the CT to the T1 MRI.
  ◦ get_surface_warp()
    ■ This method performs the sulcal-based cortical surface warping for surface electrode warping to MNI atlas space.
  ◦ get_subcort()
    ■ This method performs automatic parcellation of the subcortical structures (from freesurfer's atlas-based parcellation), and saves them to triangle-mesh.mat files for use in MATLAB or python.
  ◦ get_cvsWarp()
    ■ This method performs a combined surface-based, volumetric, and elastic warping of single subject brains to an atlas space for accurate warping of depth electrodes into common space.
  ◦ apply_cvsWarp()
    ■ This method applies the resulting warp from get_cvsWarp() to the electrode coordinates.
  ◦ checkDepthWarps()
    ■ This method produces a PDF of each depth electrode in the subject's native space and in the common atlas space for error checking of warps.
  ◦ label_elecs()
    ■ This method automatically labels electrodes based on the freesurfer atlas parcellation.
  ◦ plot_recon_anatomy()
    ■ This method plots the anatomically labeled electrodes on a surface reconstruction.
  ◦ warp_all()
    ■ This method is a wrapper method for warping the electrodes and creating pdfs for quality checking.
  ◦ plot_brain()
    ■ This method is a wrapper function for plotting the surface reconstruction and electrodes.

### Running surface reconstructions in img_pipe

Pial surface meshes are created in freesurfer, but freesurfer code is called from within img_pipe for ease of use. First we must initialize the patient object, after which we may call the appropriate methods to execute surface reconstructions.

#### Initializing the patient in img_pipe

- After AC-PC alignment as described above, start an ipython session and initialize the patient object. (Note: this process may be started in a Unix “screen” or “tmux” session if running on a remote server to avoid processes stopping after logout).
  » import img_pipe
  » subj = 'test_subj'
  » hem = 'rh'
  » patient = img_pipe.freeCoG(subj = subj, hem = hem)

#### Creating pial surface reconstructions in freesurfer

- Next, prepare the directory structure for running Freesurfer by running the following command in an ipython session:
  » patient.prep_recon()

This will create the directories elecs, mri, and mri/orig, and will copy the acpc-aligned T1.nii to mri/orig and convert it to Freesurfer mgz format.

- Next, run the following step, which will call freesurfer's recon-all script.
  » patient.get_recon()

This will create the directories bem, label, mri, scripts, src, stats, surf, tmp, touch, and trash and will run through the entire Freesurfer pipeline, which will produce a skull-stripped MRI, left and right hemisphere pial surfaces (as well as white matter and inflated surfaces), and anatomical labels for the surface and MRI files. More information on this process is provided in the Freesurfer documentation (https://surfer.nmr.mgh.harvard.edu/fswiki) and is not discussed here.

**Figure 4:**
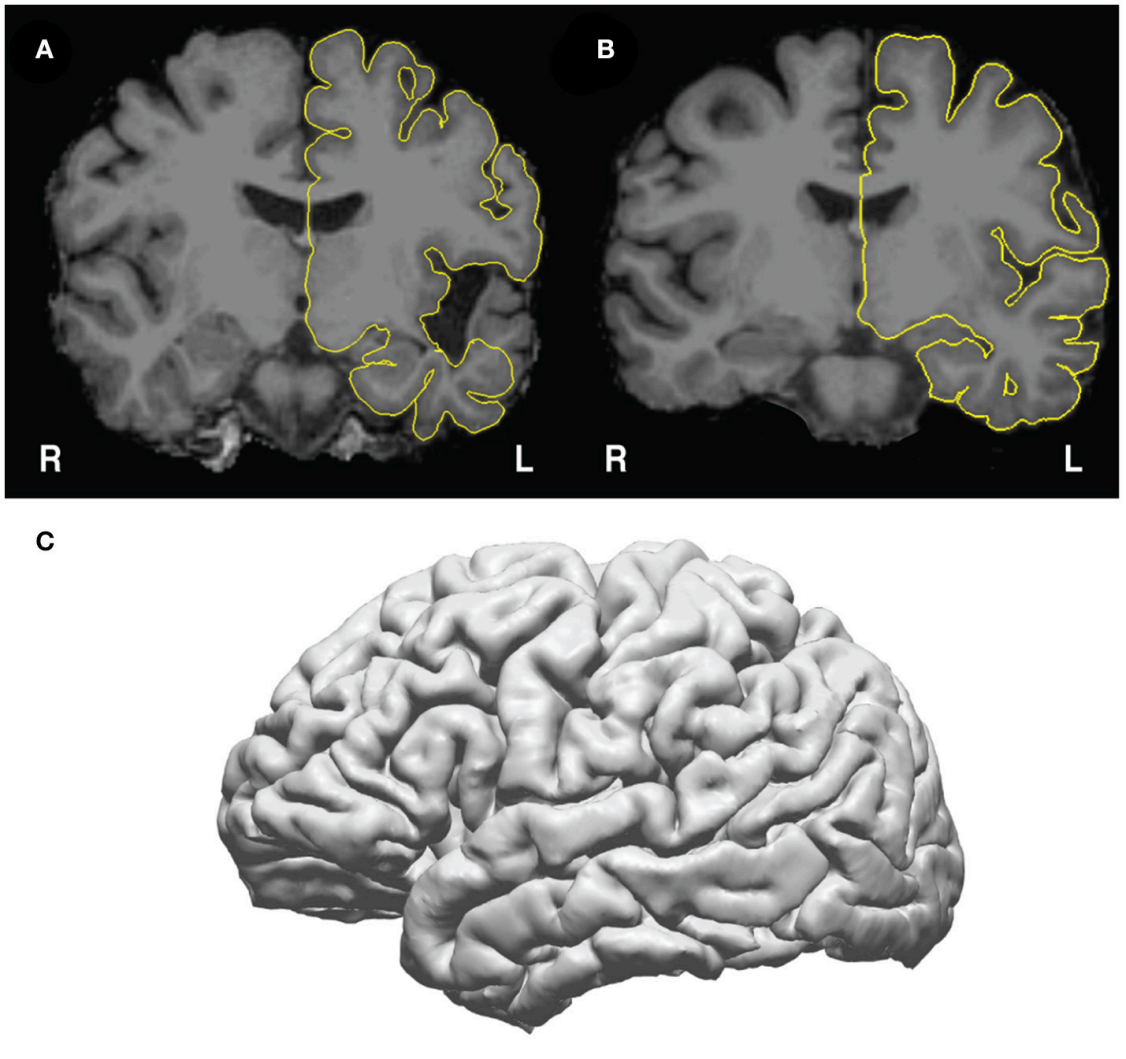
Pial surfaces identified by freesurfer during get_recon() are used to create a triangle-vertex mesh. **(A)** Example of an incorrect pial surface outline generated by freesurfer—these must be corrected by manually editing the white matter mask in freeview (see https://surfer.nmr.mgh.harvard.edu/fswiki/FsTutorial/WhiteMatterEdits_freeview for details). **(B)** Example of correctly outlined pial surface outline. **(C)** The 3D mesh generated from the pial surface from **(B)**.

- The Freesurfer pial surfaces will be in the surf directory (surf/lh.pial for left hemisphere, surf/rh.pial for right hemisphere), and the skull-stripped MRI will be in mri/brain.mgz
- **Pause point:** Check the pial surfaces in freeview (Figure 4) to assure that there is good correspondence between the pial surface and the gray/CSF boundary. To check the pial surfaces, call patient.check_pial(), which will open a Freeview window with the MRI and pial surface loaded. Scroll through the slices and check whether the pial surface accurately corresponds to the MRI. Figure 4A shows an example of a poor correspondence in the temporal lobe due to anatomical lesion – this pial surface would need to be corrected using edits to the white matter surface (https://surfer.nmr.mgh.harvard.edu/fswiki/FsTutorial/WhiteMatterEdits_freeview). Figure 4B shows good correspondence between the pial surface (yellow) and the underlying MRI. Figure 4C shows how this surface appears in the 3D view. Given a high quality T1 scan with minimal motion, the user should be able to extract a good quality pial surface that looks relatively smooth (without spiky artifacts) and that follows the underlying anatomy.
- **Note for those running code on a cluster:** The user may change the call to recon-all in get_recon() to use a queue submission procedure of choice. For example, the call to recon-all can be specified in a ‘qsub’ command to send the command to an SGE cluster computing system.

#### Converting freesurfer meshes to triangle/vertex meshes for use in MATLAB/Python

- The lh.pial and rh.pial surfaces in freesurfer contain the data for plotting a triangle/vertex mesh, which can be used later in any 3D program (for example mayavi in python, Blender, Unity, or MATLAB).
- To convert the freesurfer format to arrays of vertex coordinates (in surface RAS) and triangle indices, call the following method. By default, this will create the pial surfaces for both the left and right hemisphere.
  » patient.convert_fsmesh2mlab()
- This method can also be called with the optional keyword argument, which can also convert lh.white, rh.white, lh.inflated, and rh.inflated to triangle/vertex files.
  » patient.convert_fsmesh2mlab(mesh_name = 'inflated')
- The surface meshes created will be available as the mat files /your/Freesurfer/subjects_dir/test_subj/ Meshes/lh_pial_trivert.mat and /your/Free surfer/subjects_dir/test_subj/ Meshes/ rh_pial_trivert.mat, can now be plotted with the following command, which plots the pial meshes of both hemispheres:
  » patient.plot_brain()

#### Creation of subcortical meshes

**Figure 5:**
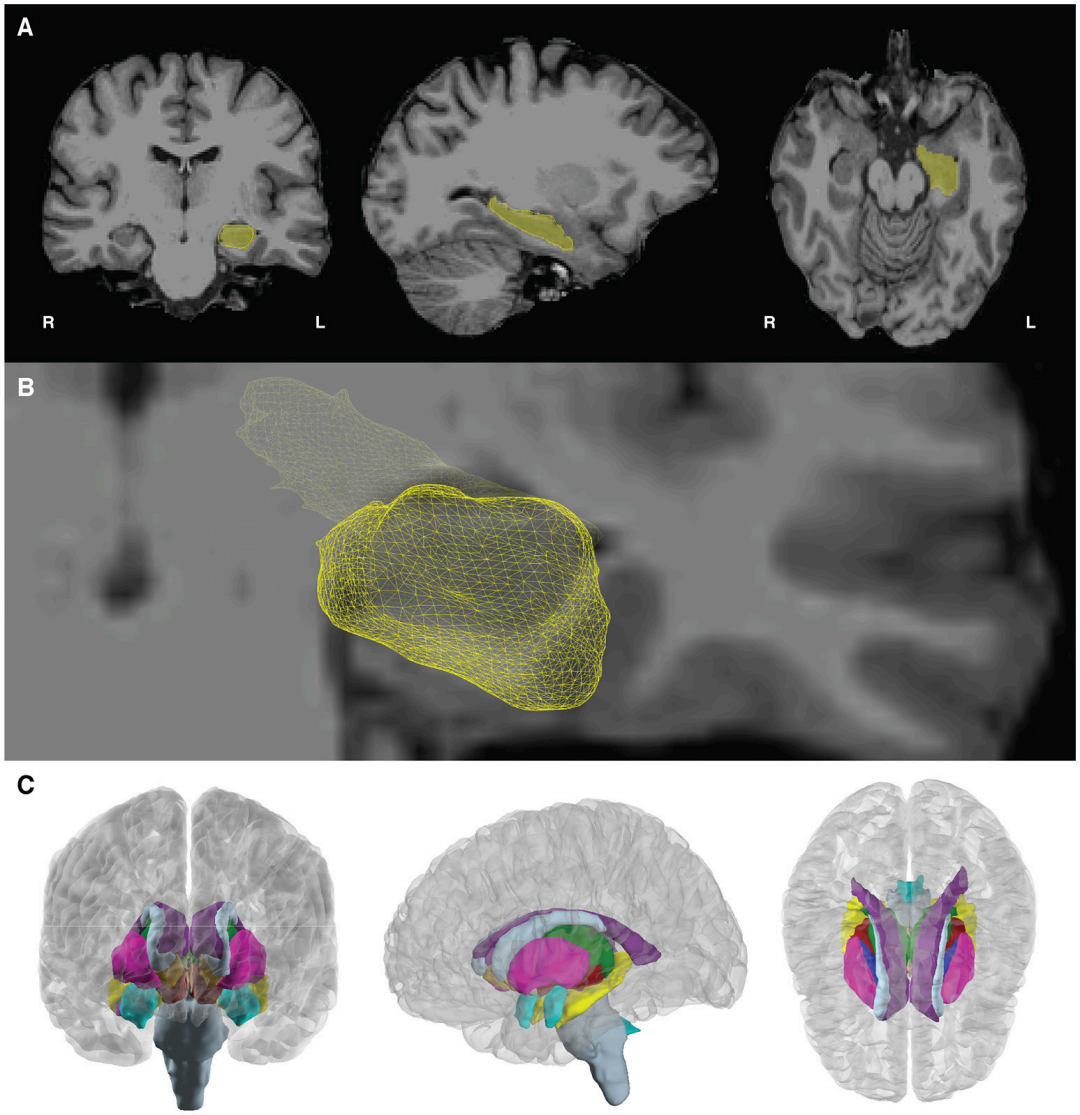
Subcortical mesh generation. **(A)** Subcortical meshes are generated from parcellating the aseg.mgz file, which assigns a numeric value to each region of interest. Labels of interest are extracted from the aseg volume, and a marching cubes algorithm is run to generate a surface mesh. In this case, the volume label for the hippocampus (yellow, number value = 17) is shown in yellow. **(B)** The resulting 3D hippocampal mesh after marching cubes, shown with a bisecting coronal slice of the medial temporal lobe. **(C)** Subcortical meshes on the template brain, cvs_avg35_inMNI152. 23 subcortical meshes in the left and right hemisphere cortical meshes are shown. Subcortical meshes are colored to match the FreeSurferColorLUT (look up table).

- In addition to the cortical pial surface meshes, the user can create surface meshes for the subcortical structures identified in freesurfer. These surface meshes are created using a script created by Anderson M. Winkler (http://brainder.org), which takes freesurfer labels (e.g., the voxels labeled as hippocampus, Figure 5A), and creates a 3D triangle mesh from these labels (Figure 5B). This method will create meshes for all subcortical structures labeled in Freesurfer (Figure 5C), including the left and right nucleus accumbens, amygdala, brain stem, caudate nucleus, ventricles (lateral, inferior lateral, third, and fourth), globus pallidus, hippocampus, putamen, thalamus, and ventral diencephalon.
  » patient.get_subcort()

- The meshes will be stored in the subjects directory within Meshes/subcortical/. These meshes can also now be viewed with the following commands:
  >>> subcort_roi = patient.roi
   (name='your_subcortical_roi')
  >>> patient.plot_brain(rois=[subcort_roi])

- To show these ROIs in conjunction with the pial surface, they can be plotted simultaneously, while controlling color and opacity.
  >>> subcort_roi = patient.roi (name =
   'your_subcortical_roi', color=(1.0, 0.0,
      0.0))
  >>> pial_roi = patient.roi (name =
   'lh_pial', opacity=0.5)
  >>> patient.plot_brain(rois=[pial_roi,
   subcort_roi])

### Co-registration of CT and MRI scans

In order to identify electrodes on the pial surface, we must transform the posteroperative CT scan into the same space as the T1 MRI. We use the normalized mutual information cost function (Ashburner and Friston, 1997) to perform this cross-modal registration in nipy.

» patient.reg_img()
  ◦ The user may also choose to specify the CT and MRI scans to align explicitly, using keyword arguments, though this is not necessary if the directory structure is set up as described. The CT scan is assumed to be in the CT directory, and the MRI scan is assumed to be in the mri directory.
  » patient.reg_img(source='CT.nii',
      target='orig.mgz')

**Figure 6:**
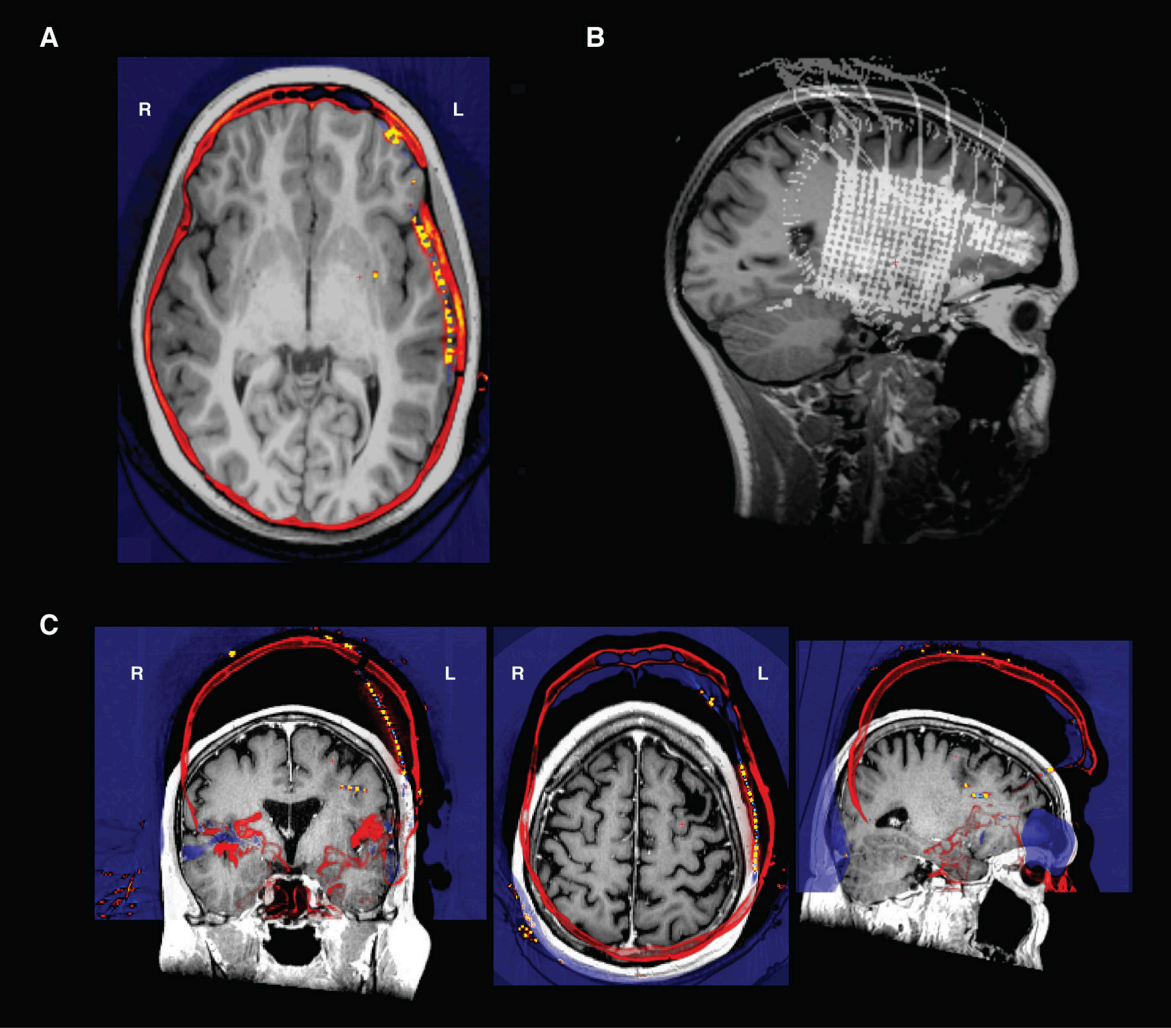
**(A)** Transverse view in Freeview of a CT and an MRI that have been co-registered- the electrodes are the orange points in the left hemisphere. Note how the skull in both the CT and MRI are aligned after registration. **(B)** An intensity projection view. **(C)** CT and T1 scan in original native space before alignment. The CT shown here (in “heat” colormap) was unable to be aligned to the T1 scan (grayscale image) because of poor initial conditions. To align, the CT was translated and rotated to be in rough alignment with the T1 scan, and then reregistered in img_pipe.

- Check the coregistration of the CT and MRI in freeview (Figure 6A). One way to do this is to load the CT on top of the MRI in the Volumes tab, then decrease the opacity of the CT to inspect the alignment of the bones in the skull. We recommend using the ‘heat’ colormap for the CT and grayscale for the MRI. Check sagittal, coronal, and axial views to ensure that the bones of the skull are aligned properly in both images. When plotting the maximum intensity projection of the CT (Figure 6B), the user should also verify that the grids and strips are in roughly the right place.
- **Note:** It is common that when the CT is aligned to the pre-op MRI, lateral grids may appear as though they are underneath the brain's surface. This is common because the placement of these grids results in a deformation of the brain surface (Hermes et al., 2010). We solve this by later projecting these electrodes to the pial surface.
- **Quality check step:** If the CT alignment fails, the user may need to change the initial alignment of the CT scan. Often, poor registrations can be traced back to poor initial conditions [T1 is not aligned to the AC-PC, or the CT scan's original position is at a very different orientation from the desired registered output (Figure 6C)]. The user may need to manually align the CT for an initial placement, then rerun the coregistration to get a precise alignment between CT and MRI). This can be done in freeview, SPM, or other neuroimaging programs.

### Manual identification of electrodes

While other methods have been developed to automatically determine the 3D spatial location of electrodes in a CT scan (LaPlante et al., 2016), in practice it is often difficult to fully automate the process given the limited resolution of most clinical CT scans, especially for high-density grids with <=4 mm spacing often employed in our research (Chang, 2015; Muller et al., 2016a,b). Here we describe the process for manually identifying electrodes that will later be automatically labeled according to their nearest anatomical location (based on Freesurfer atlas segmentation). This step also ensures that electrode numbering will match the clinical or research montage for ease of future analysis.

#### Electrode identification on the co-registered CT scan

**Figure 7:**
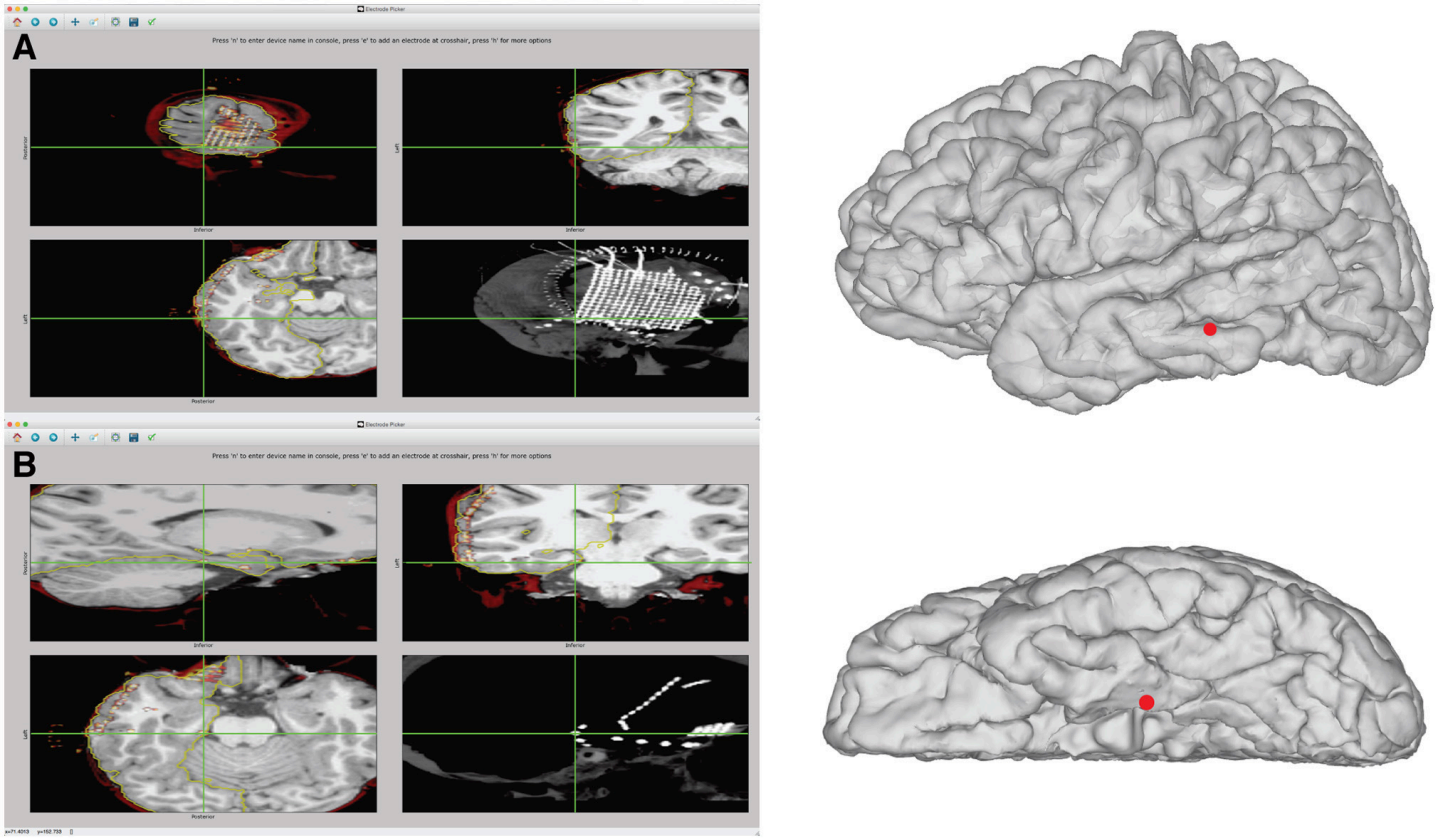
Example of identification of electrode coordinates using electrode picker. **(A)** Demonstrates the process of picking the coordinate for the most posterior inferior grid corner. On the left, the GUI is shown with the electrode selected. The pial surface, rCT, and skull stripped MRI are displayed. The upper left shows the electrode selected in the sagittal view. The upper right shows the coronal view. The bottom left shows an axial view. The lower right displays the intensity projection map of the CT, which is useful for visualizing the entire grid. To save the coordinate, press “n” to name a new device. With the center of the electrode artifact localized by the crosshairs in the axial, sagittal, and coronal views, press “e” to add a point. The coordinates are automatically saved to the “elecs” folder. The location of these points in 3D can be viewed by launching a separate 3D viewer by pressing “3.” This plot can be seen in the right panel. If the coordinates appear buried in the Mesh due to post-operative brain shift, additional steps can be taken to project the electrode to the surface, shown in Figure 8. **(B)** Example of identification of an electrode that is part of a subtemporal strip. The strip can be seen in the rCT.nii intensity projection map in the lower right panel. The coordinate is recorded from the center of the electrode artifact, seen in sagittal, coronal, and axial views. This coordinate can then be visualized on the 3D surface mesh, seen in the right panel, by typing “3.”

- Electrodes will be identified in the postoperative, co-registered CT scan using an electrode picker GUI (Figure 7). The coordinates obtained can be used to plot the electrodes on the meshes (Figure 7A, top right). To start the electrode identification process, call patient.mark_electrodes(). This will launch an interactive python GUI that will overlay the registered CT scan on top of the skull-stripped MRI. Instructions for use are detailed below. The user can also press ‘h’ (for help) while in the GUI for a list of possible commands.
- Click to navigate the crosshairs to electrode 1 of a device (a strip, depth, or grid). Numbering for depths and strips is usually distal to proximal. Intraoperative photos can be useful to verify the numbering of grids. It can also be useful to view the CT intensity projection map (bottom right subplot in the GUI, shown in Figures 7A,B) to get a sense of the arrangement of the electrodes.
- Add a new device by pressing the ‘n’ key. This will prompt the user to enter the device name in the python console (this may be behind the figure window). If marking corners of a high density grid, this could be called ‘hd_grid_corners’. If marking a hippocampal depth, this could be called ‘hippocampal_depth’. Another good heuristic is to use the labels from a provided clinical electrode montage.
- Ensure that the crosshairs are in the center of the electrode artifact in the coronal, axial and sagittal views. **Tip:** When clicking on an electrode, the user can quickly identify the correct placement in all views by clicking on an electrode in, for example, the coronal view, then zooming in to the CT scan (on a Mac, using the scroll wheel), which will re-center the other image views with the marked electrode in the center.
- Click ‘e’ to add an electrode at the crosshair position. The user should now see a colored circle in all views (sagittal, axial, and coronal) for this electrode, and a legend showing the device name will appear in the maximum intensity projection plot. Electrodes will automatically be saved to the variable ‘elecmatrix’ in a file in the elecs directory, which will be named according to the device name given by the user. Add multiple electrodes to the same device by pressing ‘e’ for each. To start adding to a new device, simply press ‘n’ to initialize a new device and enter the name into the python console and start the process as before.
- While marking electrodes, the user can zoom in and out on the plot using the mouse scroll wheel or trackpad scroll, pan with the arrow keys, and move through single slices using the “page up” and “page down” keys. They can also simply click on any of the views to move to that location in the CT and MRI.
- To change the plot view of the maximum intensity projection, choose ‘s’ for sagittal (the default), ‘c’ for coronal, or ‘a’ for axial. The maximum intensity projection is calculated for the current slice ± 15 slices, so it may be useful to scroll through this plot as well.
- The outline of the pial surface is plotted in yellow for reference. Toggle this on and off using the ‘t’ key.
- It can be helpful to plot elecmatrix on the 3D brain to check the placement of the grids and depths. To inspect a 3D visualization of the currently identified electrodes, press the number ‘3’ key. Simply close the 3D window to continue marking electrodes.
- The pial surface is created from a preoperative MRI, and postoperative brain shift can cause strips and grids in rCT.nii to appear buried within the pial surface. This shift is most noticeable in lateral grids, and can be corrected using a mean normal projection, described below. For some strips this shift is less pronounced and can be corrected manually during the electrode identification process in the electrode picker or Freeview. With lh.pial or rh.pial displayed on top of rCT, pick a coordinate near the electrode that places it on the surface of the brain. Plotting elecmatrix on the brain during this manual projection can ensure the coordinate is on the surface and looks appropriate with respect to the rest of the device.

#### Grid interpolation for high-density ECoG grids

- When using high-density (<=4 mm center-to-center spacing) grids, the resolution of the CT scan may prohibit easy identification of individual electrodes in the scan. Thus, to circumvent this issue, we use the locations of the corners of the grid and interpolate between them in evenly spaced intervals depending on the grid dimensions.
- To use grid interpolation, first identify the coordinates of the electrode grid's four corners, in channel order (for example, in a 16 x 16, 256-channel grid, corner 1 is electrode 1, corner 2 is electrode 16, corner 3 is electrode 241, and electrode 4 is channel 256). Name this file hd_grid_corners.mat and place in the elecs directory.
- In img_pipe, call the following:
  ◦ patient.interp_grid(nrows = 16, ncols
       = 16, grid_basename = 'hd_grid')
- This will create the file hd_grid_orig.mat in elecs/individual_elecs, which will then be projected to the surface in the next step.

#### Projection of subdural surface electrodes to the pial surface

**Figure 8:**
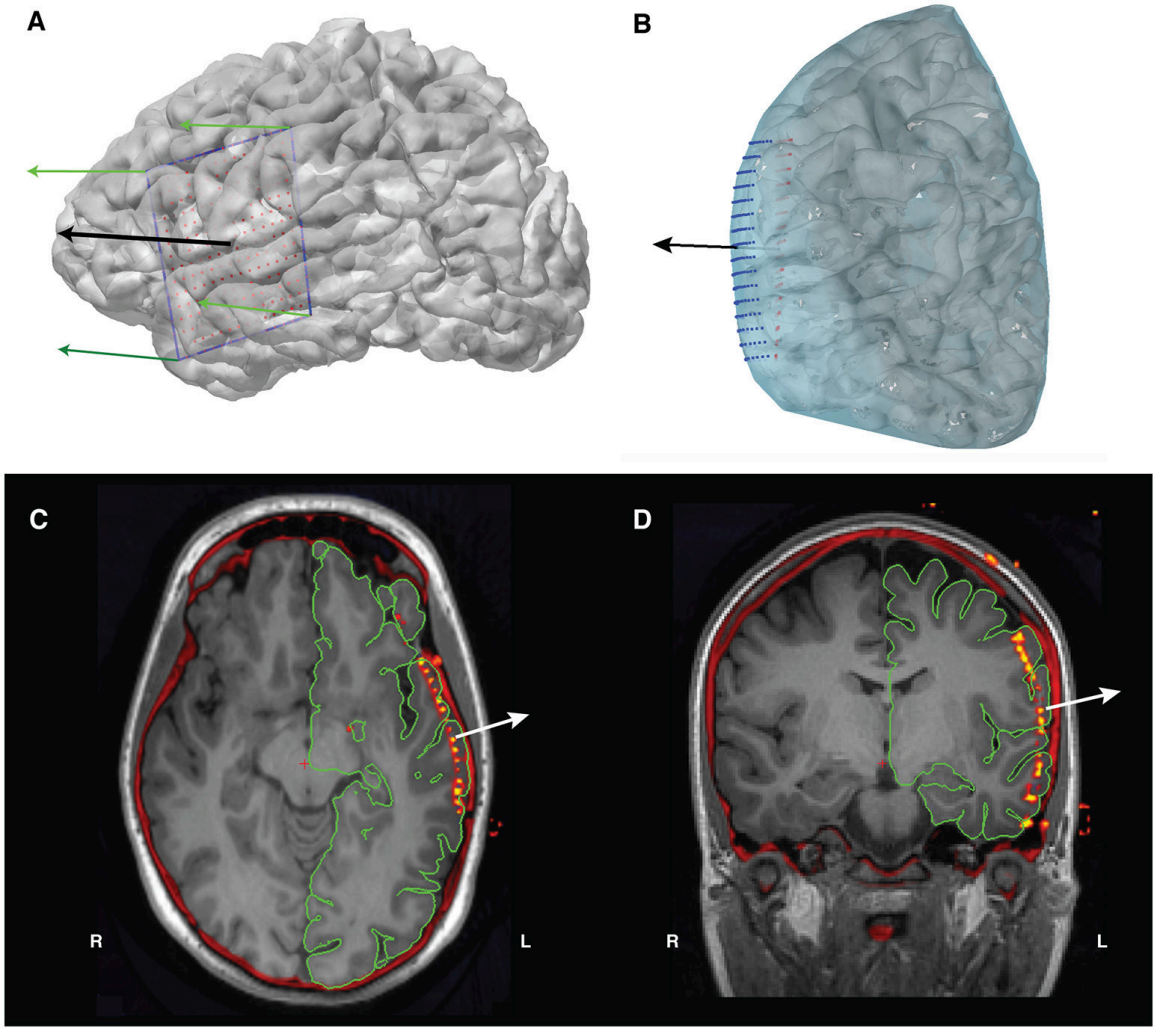
**(A)** The grid's corner electrodes are manually located. We interpolate the locations of the rest of the grid electrodes using these corner coordinates, giving us the electrode grid shown in red. The green arrows are the four normal vectors calculated from the corners, and the black arrow is the mean of those normal vectors and will act as our projection direction. **(B)** Projection of the interpolated grid (red) to the convex hull of the pial surface (blue) using the mean normal vector (black arrow). The final projected electrode grid is shown in blue. **(C)** Axial and **(D)** coronal views of the co-registered CT overlaid on the T1 MRI, showing the location of the electrodes prior to surface projection.

- To project the electrodes to the pial surface, we use the four corner electrodes (Figure 8A) of the grid to obtain a set of four vectors that outline the grid. Using these outline vectors, we can obtain a set of four normal vectors, one corresponding to each corner (Figure 8B). We then take the mean of these vectors to obtain a mean normal vector, which will be the projection direction for the unprojected interpolated electrode grid. Using this mean normal vector, we then project every electrode in the interpolated grid outwards to the smoothed dural surface of the pial mesh (Figures 8C,D). Note that, when projecting an orbitofrontal grid, the dural surface of the cortical mesh without temporal lobe is used to ensure projection to the bottom surface of the orbitofrontal cortex.
- patient.project_electrodes(elecfile_prefix = 'hd_grid')
- To project a grid on the orbitofrontal cortex, use:
  patient.project_electrodes(elecfile_
  prefix = 'OFC_grid', surf_type = 'OFC')
- To visualize these electrodes on the brain, use the plot_brain method:
  >>>grid_elecs =patient.get_elecs
  (elecfile_prefix='hd_grid')['elecmatrix']
  >>>patient.plot_brain(elecs=grid_
  elecs)

#### Creation of the elecs_all.mat file

- The elecs_all.mat file combines the individual device coordinates to represent the electrodes in the recording montage order. elecs_all.mat contains elecmatrix and eleclabels. elecmatrix contains the x, y, z coordinates of each device, combined in the appropriate montage order. eleclabels contains device descriptors corresponding to these devices, shortened device ID (e.g. G1) in column 1, long device ID (e.g. L256GridElectrode1) in column 2, and device type (e.g. grid) in column 3. The possible values for “device type” should be ‘grid’, ‘strip’, or ‘depth’. Short and long device ID names should be unique. For an example of what this will look like, see Table 1.
- After creating the.mat files for each electrode device in patient.mark_electrodes(), initialize ‘elecs_all.mat’ by calling patient.make_elecs_all(), which will prompt the user for the information mentioned above, and automatically create the ‘elecs_all.mat’ file.

**Table 1:** Example structure of the anatomy variable in elecs_all.mat.

**Shortened device ID**	**Long device ID**	**Device type**	**Anatomical location**
G1	L256GridElectrode1	grid	superiortemporal
G2	L256GridElectrode2	grid	superiortemporal
…	…	…	…
G256	L256GridElectrode256	grid	rostralmiddlefrontal
AD1	LAmygdalaDepth1	depth	Left-hippocampus
AD2	LAmygdalaDepth2	depth	Left-hippocampus
AD3	LAmygdalaDepth3	depth	Left-amygdala
AD4	LAmygdalaDepth4	depth	Left-amygdala
AD5	LAmygdalaDepth5	depth	Left-amygdala
AD6	LAmygdalaDepth6	depth	Left-amygdala

### Automated anatomical labeling of electrodes

- To automatically label the electrodes, call the label_elecs() method:
  » patient.label_elecs(elecfile_prefix
  ='elecs_all', atlas_surf='desikan-
  killiany, atlas_depth='destrieux)
- This will add a fourth column to the elecs_all.mat file that will match labels from the Desikan-Killiany atlas (Desikan et al., 2006) or the Destrieux atlas (Fischl et al., 2004). The Desikan-Killiany atlas is a coarser parcellation that we generally use for surface labeling, whereas the more complex Destrieux atlas is used for labeling depth electrodes.
- For surface electrodes, labeling is performed by finding the closest surface vertex (according to Euclidean distance) and determining the Freesurfer label of that point from the annotation file associated with the atlas of interest. For depth electrodes, the label is assigned according to the voxel label in the parcellated volume (aparc+aseg.mgz or aparc.a2009s+aseg.mgz).

#### Pause point: quality checking anatomical labeling

**Figure 9:**
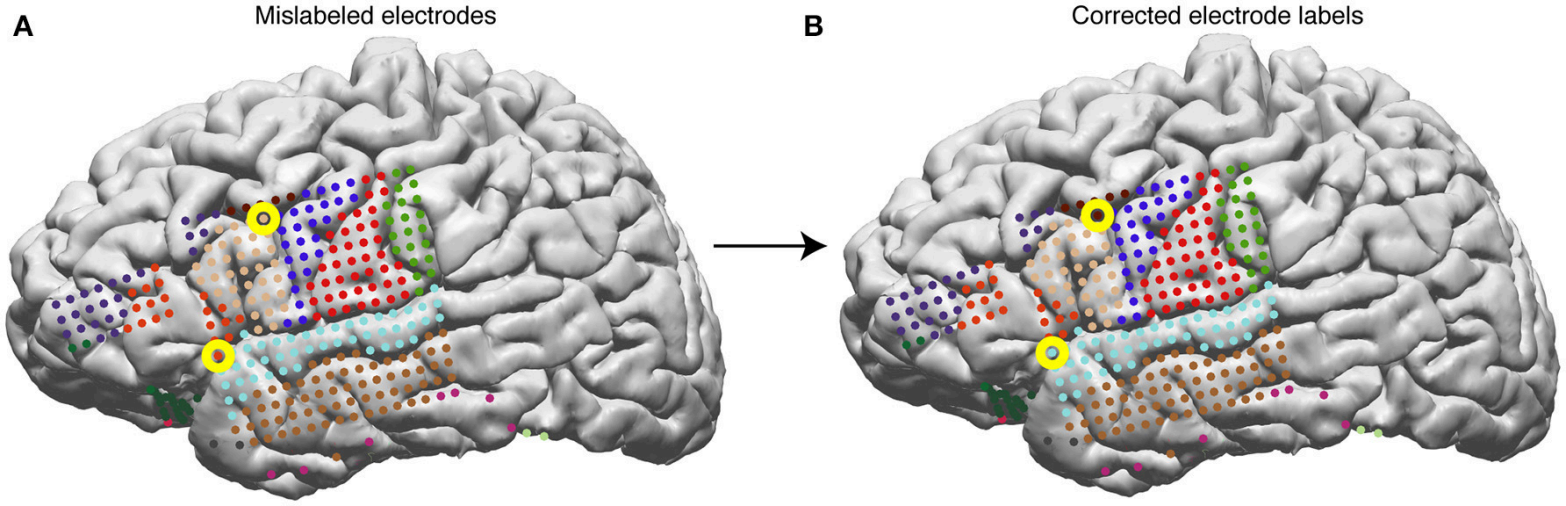
Surface electrodes are labeled by Freesurfer using the Desikan-Killiany atlas—each color represents a different anatomical region. Electrodes are labeled using the anatomical label of the nearest vertex in the brain mesh. **(A)** Anatomical labeling of surface electrodes with errors identified as yellow circles. **(B)** Corrected anatomical labeling.

- Quality checking the anatomical labeling is done using the method plot_recon_anatomy().
   » patient.plot_recon_anatomy()
- The automatic labeling will sometimes mislabel electrodes that lie on the border between two areas. For example, in Figure 9A, an electrode on superior temporal gyrus (label: superiortemporal) is labeled pars triangularis (label: parstriangularis), and an electrode on the caudal middle frontal gyrus is labeled pars opercularis.
- To correct an anatomical label, use the edit_elecs_all() method. The arguments include label names as keys and lists of electrode numbers as values, and the prefix of the electrode file. For example, to correct the two mislabeled electrodes in Figure 9, the user would perform the following:
  ◦ revision_dict =
      {'superiortemporal':[246],
    'caudalmiddlefrontal:[174]}
  ◦ patient.edit_elecs_all(revision_dict,
      elecfile_prefix = 'elecs_all')

### Warping surface and depth electrodes to a common atlas

To warp electrodes from the native space to a common atlas space, call patient.warp_all(elecfile_prefix = 'elecs_all'), which will by default warp the subject brain to the cvs_avg35_inMNI152 brain, and generate the warped coordinates for both surface and depth electrodes in elecs_all.mat. Options passed into this method may be changed if the user wishes to warp a subset of electrodes, such as only surface or only depth electrodes, or if they wish to use a different template brain.

**Figure 10:**
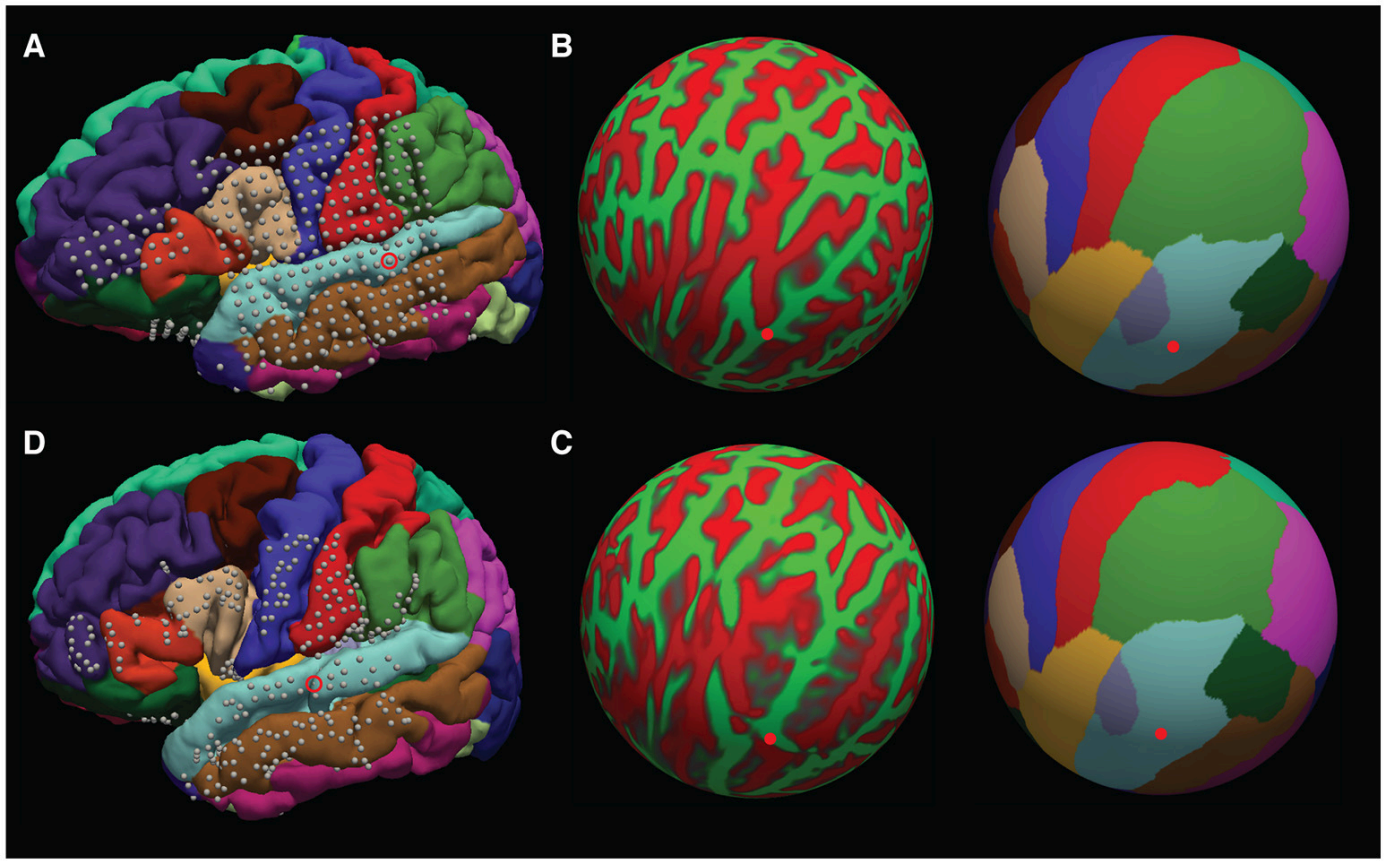
Surface warping procedure. **(A)** Electrodes on native brain. Gyri are colored according to anatomical designation by Freesurfer. An example electrode localized to the native STG can be seen circled. Electrodes are warped from the subject's native brain to the cvs_avg35_inMNI152 average brain in spherical surface space. The native lh.sphere is shown in **(B)**, with the location of the same STG electrode marked in red. lh.sphere is shown with curvature and anatomical color coding. Warping occurs when the native lh.sphere is warped to match the lh.sphere for the cvs_avg35_inMNI152 average brain, shown in **(C)**. The same STG electrode is now shown in red on the average brain. Finally, the localization in spherical space is used to localize the electrode on the pial surface of the cvs_avg35_inMNI152 average brain, shown in **(D)**.

The surface warps are generated by projecting the pial surfaces of the subject and template brains into a spherical coordinate space, and aligning the surfaces in that space – this is shown in Figures 10A–D. Depth warping is performed using a combination of volumetric and surface warping (Postelnicu et al., 2009). We have found that surface warping the strip and grids results in more accurate placement of the warped electrodes on the same gyri as in the native space, whereas for depth electrodes a volumetric and surface warping is necessary.

Note that warping depth electrodes takes significantly longer than warping surface electrodes.

#### Pause point: quality checking warps

- patient.warp_all() will have generated PDFs in the subject's elecs directory (^*^_recon_anatomy.pdf and ^*^_warped_recon_anatomy.pdf) of the electrodes and their warps. To check the warp interactively, use patient.plot_recon_anatomy_compare_warped(template = 'cvs_avg35_inMNI152', elecfile_prefix = TDT_elecs_all).

#### Pause point: quality checking depth electrode warping

**Figure 11:**
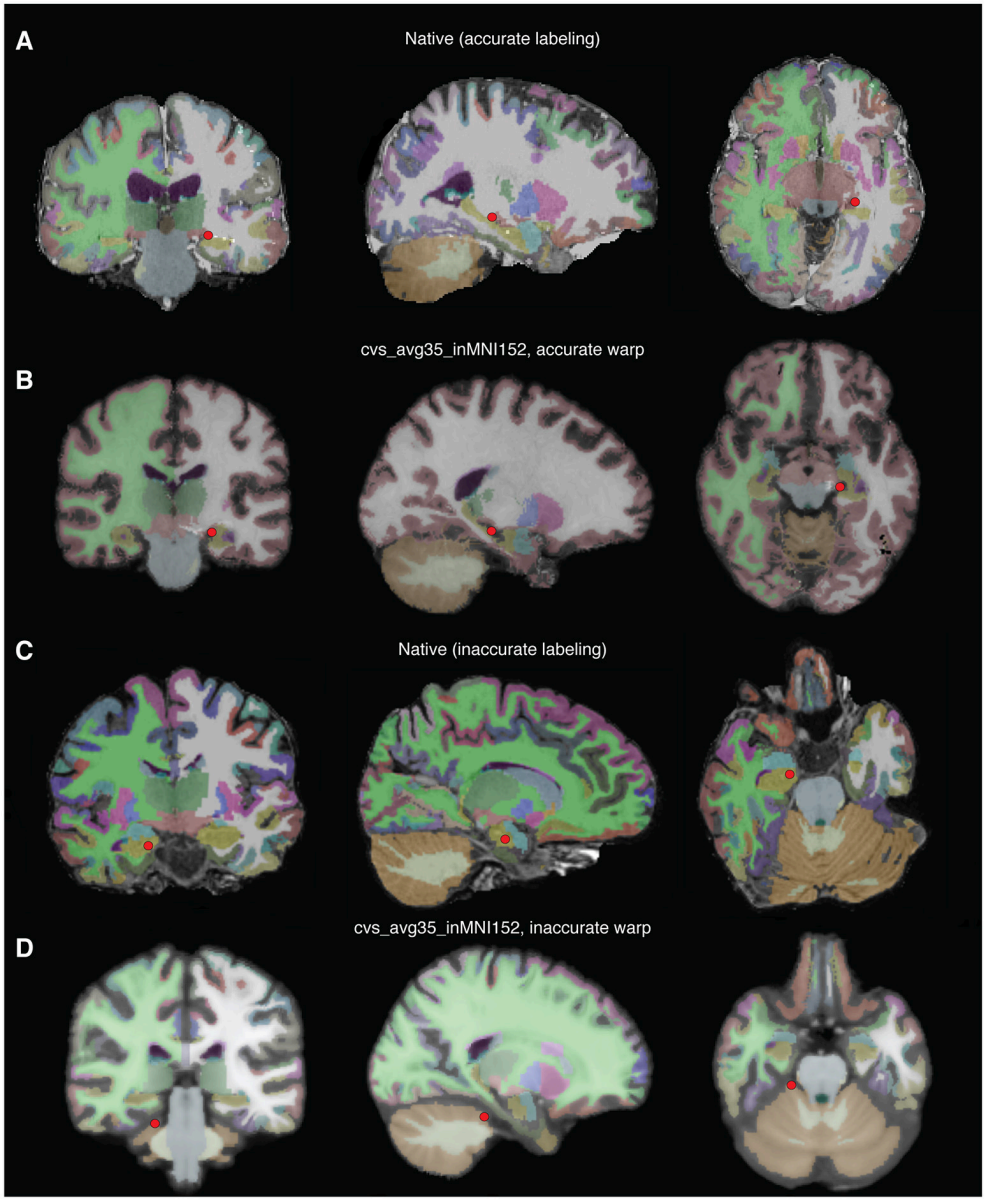
Examples of accurate depth warp **(A,B)** and inaccurate depth warp **(C,D)**. Colors correspond to anatomical label, and the red circle marks electrode in single subject brain **(A,B)** and the location it is warped to in the CVS brain **(C,D)**. The electrode of interest in **(A)** retains its anatomical label, left hippocampus, when warped to the CVS brain in **(B)**. However, the electrode of interest in **(C)** is incorrectly warped from right hippocampus in the single subject brain to cerebellar cortex in the CVS brain **(D)**.

- patient.check_depth_warps() will generate a pdf in the subject's elecs directory, depthWarpsQC.pdf comparing an electrode's original location in the subject brain compared to its warped location in the template brain. Figures 11A,B shows an accurate warp, and Figures 11C,D shows an inaccurate warp. If an electrode's warped location is inaccurate, either remove the electrode from the warped electrode coordinate matrix, or manually choose the location in the template brain.

### Plotting electrodes and activity on the pial surface

**Figure 12:**
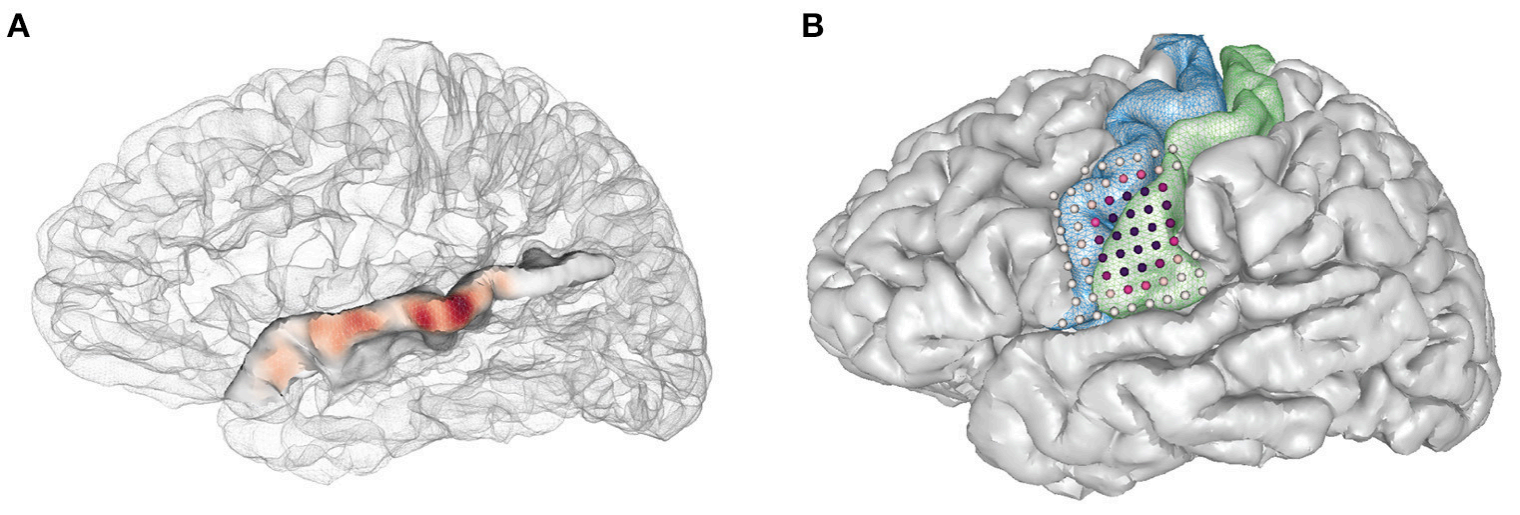
Examples using the plot_brain() function, **(A)** Gaussian representation of electrode weights on a superior temporal gyrus ROI, red-blue color map. **(B)** simulated electrode weights on precentral and postcentral ROIs represented as colored spheres, colored using a red-purple color map.

patient.plot_recon_anatomy() plots anatomically labeled electrodes on the brain.
• Warped electrodes can be plotted on a template brain by calling patient.plot_recon_anatomy(elecfile_prefix = 'warped_elecs_file', template = your_template).
• To plot electrode activity on the brain's surface, use the method plot_brain(). The user can control the opacity of the brain mesh, wireframe/surface representation, electrode colors, and colormap. For example:
  >>> pial = patient.roi('pial', color=
    (0.6,0.3,0.6), opacity=0.1, representation
    ='wireframe', gaussian=True)
   >>> hipp = patient.roi('lHipp', color=
    (0.5,0.1,0.8), opacity = 1.0,
    representation='surface', gaussian=True)
   >>> elecs = patient.get_elecs()
    ['elecmatrix']
   >>> patient.plot_brain(rois=[pial,hipp],
    elecs=elecs, weights=np.random.uniform
    (0,1,(elecs.shape[0])))
• The following code will allow the user to show activity on the superior temporal gyrus as interpolated gaussian blobs (Figure 12A):
    >>> elecmatrix = patient.get_elecs(roi =
         'superiortemporal') ['elecmatrix']
    >>> pial = patient.roi('lh_pial',
         color=(0.8,0.8,0.8), opacity=0.2,
         representation=wireframe,
         gaussian=False)
    >>> patient.make_roi_mesh
         ('superiortemporal',
         label_list=['superiortemporal'])
    >>> stg_roi = patient.roi
         ('lh_superiortemporal',
         color=(0.3,0.6,0.8), opacity=1.0,
         representation='surface',
         gaussian=True)
    >>> patient.plot_brain(rois =
         [pial,stg_roi], elecs=elecmatrix,
         weights=np.random.uniform(−1,0,
         elecmatrix.shape[0]), showfig=True,
         screenshot=True, cmap='RdBu')
  Here's another example, plotting regions of interest (ROIs) for the pre- and postcentral gyri as well as electrode activity (Figure 12B).
    >>> #get all coordinates of elecs in
         precentral+postcentral
    >>> elecmatrix = np.concatenate
         ([patient.get_elecs(roi=
         'postcentral')['elecmatrix'],
         patient.get_elecs(roi ='precentral')
         ['elecmatrix']],axis=0)
    >>> #get meshes of precentral,
         postcentral gyri, and pial surface
   
    >>> patient.make_roi_mesh('precentral',
         label_list=['precentral'])
   
    >>> patient.make_roi_mesh('postcentral',
         label_list=['postcentral'])
   
    >>> precentral_roi = patient.roi
         ('lh_precentral',
         color=(0.3,0.6,0.8), opacity=1.0,
         representation='wireframe',
         gaussian=False)
   
    >>> postcentral_roi = patient.roi
         ('lh_postcentral',
         color=(0.5,0.8,0.5), opacity=1.0,
         representation='wireframe',
         gaussian=False)
   
    >>> pial = patient.roi('lh_pial',
         (0.8,0.8,0.8),1.0,'surface',False)
   
    >>> #calculate distances from two random
         electrodes' coordinates.
   
    >>> distance1 = np.array
         (map(np.linalg.norm, elecmatrix-np.tile
         (elecmatrix[30,:], (67,1))))
   
    >>> distance2 = np.array
         (map(np.linalg.norm, elecmatrix-np.
         tile(elecmatrix[20,:], (67,1))))
   
    >>> patient.plot_brain
         (rois=[pial,sub_roi,sub_roi2],
         elecs=elecmatrix,
         weights=scipy.stats.zscore
         (distance1 + distance2),
         showfig=True,
         screenshot=True,
         cmap='RdPu')

### Other notes

- It is possible to launch specific sub-methods of img_pipe in separate python sessions. For example, the user may wish to launch patient.get_recon() 1 day, then open another python session later for patient.mark_electrodes(). To do this, simply reinitialize the patient according to instructions in section Initializing the Patient in img_pipe, and then continue with the next step.
- If the user prefers not to AC-PC orient the images to start (and assuming that this does not affect the quality of the warping step), it is possible to reorient the final surface and electrode.mat files using Freesurfer's talairach.xfm file, which will put them into a common orientation. To do this, the user could perform the following step on the native space electrodes and surface:
  ◦ patient.apply_xfm
     (xfm_dir='mri/transforms',
     xfm_file='talairach.xfm',
     source_file='elecs/TDT_elecs_all.mat',
     target_file='elecs/
     TDT_elecs_all_2tal.mat',
     file_type='elecs')
  ◦ patient.apply_xfm
     (xfm_dir='mri/transforms',
     xfm_file='talairach.xfm',
     source_file='Meshes/
     rh_pial_trivert.mat',
     target_file='Meshes/
     rh_pial_trivert_2tal.mat',
     file_type='surf')
  ◦ patient.apply_xfm
     (xfm_dir='mri/transforms',
     xfm_file='talairach.xfm',
     source_file='Meshes/
     lh_pial_trivert.mat',
     target_file='Meshes/
     lh_pial_trivert_2tal.mat',
     file_type='surf')

## Discussion and conclusion

Here we have described a full pipeline for obtaining high quality 3D surface renderings, labeled, localized electrodes, and atlas-warped electrodes from an input T1 and CT scan. This process has been optimized from start to finish to allow the user to easily create the needed files for later functional analyses. Our software relies on completely free, easily downloadable, open source tools. We also provide plotting tools so that users may easily plot localized electrodes in either the native subject space or a warped subject space.

### Common problems/troubleshooting

In previous sections we described some pause points for quality checking in between each of the steps in this protocol. However, for convenience we also provide here some potential pitfalls that users may encounter while running this pipeline.

#### Installation issues

Most installation issues can be avoided by following the instructions above and using the conda environment to install the required packages. Using the conda environment avoids potential version conflicts (for example VTK 6.3.0 is required when using Python 2.7, but VTK 7.0.0 is required for Python 3.5. In addition, pyqt version 4 is required rather than the most recent version 5). Be sure that environmental variables are set for FREESURFER_HOME and SUBJECTS_DIR, and that paths are set appropriately. Note that Freesurfer does not currently run on Windows, so for users running Windows we suggest running a Linux virtual machine with VirtualBox or other virtualization software.

If issues are encountered when importing img_pipe despite following the conda environment instructions, the user should attempt the import outside the img_pipe directory. Check that the version imported is located in a directory such as ~/anaconda/lib/python2.7/site-packages/img_pipe.

#### AC-PC alignment issues

The user may find that aligning scans in Freeview causes part of the volume to be cut off, especially where the image itself must be translated over a large distance (more than a centimeter or two). We have found that this issue is most common with Freeview v1.0, and does not occur with Freeview v2.0.

#### Issues with freesurfer reconstruction

If the freesurfer reconstruction fails or is of poor quality, there are a number of potential reasons. The most common in our experience are (1) poor image contrast due to scanner parameters or due to subject motion, (2) poor image resolution (greater than 1 × 1 × 1 mm voxel size), and (3) images with GAD contrast used in clinical procedures. In all of these cases, the best course of action is to obtain a better scan or see if one is already available. Suggestions for scanning parameters are available on freesurfer's website. However, if no other scans are available, it is usually better to use a high resolution scan with GAD contrast than a low resolution scan without contrast. The surface reconstruction will likely include some defects where the blood vessels interfere with good gray/white matter boundary detection, but the surfaces are usually acceptable, if mediocre. For subjects with large lesions or tumors, manual segmentation may be necessary and is not described here.

#### Problems with CT to MRI coregistration

For appropriate CT to MRI coregistration, the user should select the highest resolution T1 and CT scans, both without contrast. The initial conditions of these two scans may also influence whether good coregistration is achieved. As mentioned previously, if the coregistration step fails, the CT and the T1 scan should first be roughly aligned (this can be done in Freeview, SPM, or other neuroimaging programs), and then coregistration re-attempted. Other parameters that can be adjusted in the coregistration method are the smoothing parameter, interpolation method, and tolerance parameters for function minimization. These parameters are described in the docstring for img_pipe.reg_img.

#### Problems with manual electrode localization

At times, relatively poor resolution of a CT scan may make it difficult to localize individual electrodes as they tend to blur into one another. In this case, the user may opt to identify the first and last electrodes in a depth electrode, for example, and linearly interpolate between them. This is not currently implemented directly in img_pipe, but can be performed in python with minimal effort.

Other issues with identifying electrodes can be aided by using the 3D viewer [press ‘3’ while in patient.mark_electrodes() mode]. This can help the user to determine whether they are correctly identifying a strip electrode curving around the temporal lobe, or whether they have correctly labeled two depth electrodes that cross one another.

#### Problems with electrode projection

One common problem with electrode projection is that electrodes will be projected to the wrong hemisphere. In this case, the problem is that the hemisphere of implantation was set incorrectly when initializing the patient.

Another potential problem can occur when projecting electrodes to the bottom surface of the brain (for example, subtemporal electrodes), since the surface may be concave and electrodes may appear to be “off” of the brain. In this case, we suggest using the smoothed dural surface rather than the convex hull for projection. In addition, we provide the option of projecting to an orbitofrontal ROI (with the option elecfile_prefix = 'OFC_grid', see section Projection of Subdural Surface Electrodes to the Pial Surface), which can circumvent problems where the temporal lobe interferes with a normal projection.

#### Problems with electrode labeling

Poor electrode labeling is usually a result of poor image quality and thus poor segmentation of the T1 scan by freesurfer. It is best to quality check the results of the pipeline at each step, so that the freesurfer labeled segmentation (aseg.mgz) nicely follows the anatomy of the T1. If this is not the case, you may need to start over with a new T1 scan with better gray/white matter contrast, or if this is not possible, manually correct any labels that were classified incorrectly.

#### Problems with electrode warping

Electrode warping issues may be encountered when using electrodes that have been projected to the convex hull. This is because, prior to warping, electrodes must be snapped to their nearest surface vertex. Sometimes the closest surface vertex ends up moving the electrode to the incorrect gyrus (for example, an STG electrode might end up above the Sylvian Fissure). If this occurs, the best course of action is to create a temporary file with the electrode coordinates, manually correcting the electrode location to the closest surface vertex on the correct gyrus. Then, perform the warp using this input file (change elecfile_prefix to the temporary name).

#### Other problems

Other problems may be encountered if the user incorrectly enters the hemisphere of implantation (patient.hem must be set to “lh” or “rh” if surface grids are used, otherwise if a stereo EEG case with no grids, “stereo” will suffice).

While this software provides an easy way to perform electrode localization, it is not without the common caveats inherent to all surface ECoG research. In particular, deformation of the brain surface results in a nonlinear warping of the brain's surface (Hermes et al., 2010; Dykstra et al., 2012), which means that even with perfect co-registration of a post-operative CT to a pre-operative MRI, the localization of electrodes must be verified independently with intraoperative photos when possible, and localization should be reconciled with recorded functional properties.

### Future work

In future work, we hope to incorporate automated identification of surface electrodes to the pipeline. We have found that current algorithms work well for low-density grids (LaPlante et al., 2016), but often fail for grids with 4 mm or less pitch. The failure modes of automated electrode localization packages often result in labor-intensive correction procedures (e.g,. removing false positives, reordering electrodes so they are in the correct montage order). We have found this error correction to be slower than manual electrode identification by an experienced user, thus here we present only manual methods, although ideally the full pipeline would be automated. It is possible that with improvements in electrode detection algorithms, coupled with higher resolution CT scans, a fully automated solution would be possible.

Despite these limitations, our software is free, flexible, easy to use, and we hope will provide a way for ECoG labs to create automatically labeled and warped electrodes for ease of future analysis.

## Author contributions

LH, DC, ML, and EC conceived of the work; LH, DC, and ML performed analysis; LH and DC wrote the analysis code; all authors contributed to drafting and revising the article and approved of the final version.

### Conflict of interest statement

The authors declare that the research was conducted in the absence of any commercial or financial relationships that could be construed as a potential conflict of interest.
